# Project20: Does continuity of care and community-based antenatal care improve maternal and neonatal birth outcomes for women with social risk factors? A prospective, observational study

**DOI:** 10.1371/journal.pone.0250947

**Published:** 2021-05-04

**Authors:** Hannah Rayment-Jones, Kathryn Dalrymple, James Harris, Angela Harden, Elidh Parslow, Thomas Georgi, Jane Sandall

**Affiliations:** 1 Department of Women and Children’s Health, Faculty of Life Sciences & Medicine, King’s College London, London, United Kingdom; 2 Clinical Research Facility, Chelsea and Westminster NHS Foundation Trust, London, United Kingdom; 3 School of Health Sciences, City University of London, London, United Kingdom; 4 St Mary’s Hospital, Imperial College NHS Trust, London, United Kingdom; 5 School of Population Health & Environmental Sciences, Faculty of Life Sciences & Medicine, King’s College London, London, United Kingdom; Western Sydney University, AUSTRALIA

## Abstract

**Background:**

Social factors associated with poor childbirth outcomes and experiences of maternity care include minority ethnicity, poverty, young motherhood, homelessness, difficulty speaking or understanding English, migrant or refugee status, domestic violence, mental illness and substance abuse. It is not known what specific aspects of maternity care work to improve the maternal and neonatal outcomes for these under-served, complex populations.

**Methods:**

This study aimed to compare maternal and neonatal clinical birth outcomes for women with social risk factors accessing different models of maternity care. Quantitative data on pregnancy and birth outcome measures for 1000 women accessing standard care, group practice and specialist models of care at two large, inner-city maternity services were prospectively collected and analysed using multinominal regression. The level of continuity of care and place of antenatal care were used as independent variables to explore these potentially influential aspects of care. Outcomes adjusted for women’s social and medical risk factors and the service attended.

**Results:**

Women who received standard maternity care were significantly less likely to use water for pain relief in labour (RR 0.11, CI 0.02–0.62) and have skin to skin contact with their baby shortly after birth (RR 0.34, CI 0.14–0.80) compared to the specialist model of care. Antenatal care based in the hospital setting was associated with a significant increase in preterm birth (RR 2.38, CI 1.32–4.27) and low birth weight (RR 2.31, CI 1.24–4.32), and a decrease in induction of labour (RR 0.65, CI 0.45–0.95) compared to community-based antenatal care, this was despite women’s medical risk factors. A subgroup analysis found that preterm birth was increased further for women with the highest level of social risk accessing hospital-based antenatal care (RR 3.11, CI1.49–6.50), demonstrating the protective nature of community-based antenatal care.

**Conclusions:**

This research highlights how community-based antenatal care, with a focus on continuity of carer reduced health inequalities and improved maternal and neonatal clinical outcomes for women with social risk factors. The findings support the current policy drive to increase continuity of midwife-led care, whilst adding that community-based care may further improve outcomes for women at increased risk of health inequalities. The relationship between community-based models of care and neonatal outcomes require further testing in future research. The identification of specific mechanisms such as help-seeking and reduced anxiety, to explain these findings are explored in a wider evaluation.

## Background and rationale

Health inequalities across the globe are influenced by social factors such as poverty, social deprivation, isolation, oppression, and discrimination. The large disparities seen in birth outcomes within high income countries often reflects their socioeconomic gradient, with mortality rates closely linked to disadvantages related to poverty, ethnicity, age and other social factors [1–6]. For example, the maternal mortality rate is disproportionality high for African American and Hispanic women in the US [7], Black, Asian and minority ethnic women in the UK [6, 8, 9], refugee and migrant women in other parts of Europe [10, 11], and Aboriginal and Torres Strait Islander women in Australia [12]. It is difficult to summarise the impact of specific social risk factors on inequalities in birth outcomes due to the nature of intersecting factors, and the accumulation of risk associated with poverty and ethnicity. Table 1 below presents an overview of social risk factors that are associated with poor birth outcomes and exacerbate health inequalities in high income countries. Pregnancies of women with these social risk factors are over 50% more likely to end in stillbirth or neonatal death, and are associated with increased rates of miscarriage, termination, premature birth, low birth weight, caesarean section, and maternal death [6, 8, 13–23].

**Table 1:** Social risk factors associated with poor perinatal outcomes and experiences of maternity care [6, 7, 14, 18, 24–33].

Women who find services hard to access Women needing multi-agency services	
Black and Minority ethnicity	Mental health
Social isolation	Safeguarding concerns
Poverty/Deprivation/Homelessness	Substance and/or alcohol abuse
Refugees/Asylum seekers	Physical/emotional and/or learning disability
Non-native language speakers	Female genital mutilation
Victims of abuse	HIV positive status
Sex Workers	
Young Mothers	
Single Mothers	
Travelling community	

The UK ranks 22^nd^ in maternal mortality for OECD countries [34], and 19^th^ for infant mortality in Europe [35]. The London maternal mortality thematic review [15] found that over half of the 22 women who died in London in 2017 were from a Black or minority ethnic background, many had multiple complex social, medical and mental health factors. The review found that for the majority of maternal deaths there were missed opportunities to correctly diagnose and treat complications due to barriers across the maternity care pathway. The reviews recommendations are in line with the World Health Organisations stance on improving maternity care: *‘To improve maternal health*, *barriers that limit access to quality maternal health services must be identified and addressed at both health system and societal levels’* [36].

There is a strong evidence base that good quality midwifery care, particularly when it involves continuity of care, leads to improved outcomes for women and children and the unnecessary use of interventions in high income countries [37, 38]. Midwife-led continuity of care is defined as when "the midwife as the lead professional in the planning, organisation and delivery of care given to a woman from initial booking to the postnatal period" [39]. Although the Cochrane review of models of midwifery care [40] found that women who received continuity of care had improved birth outcomes, fewer preterm births, fetal loss and neonatal death than those receiving standard maternity care, it did not report on whether outcomes differed for women with social risk factors. The authors recommended that future research should explore this population and address the underlying mechanisms of the improved outcomes. For example, whether the observed benefits can be attributed to the quality of the relationship between the midwife and woman, or other factors such as place of care. Other specialist models of maternity care, for example group antenatal care such as ‘centring pregnancy’ and ‘pregnancy circles’, and family nurse partnerships are currently being trialled to explore their impact on outcomes for women with social risk factors [41–45]. It is hypothesised that culturally safe and community-based models of care which adopt a life course approach might help to reduce maternal and neonatal health inequalities, enhance care and improve women’s experiences of maternity care [46–48]. This impact of these place-based aspects of maternity care is poorly understood and under-researched, particularly in the UK context and for women with social risk factors who are more likely to be socially isolated and struggle to integrate with their local community.

There are a number of services across the UK providing ‘specialist care’ to women with social risk factors that often incorporate continuity of care in community settings and aim to reduce health inequalities, but they are under evaluated and often vulnerable to organisational restructuring [49]. Recent UK policy [50, 51] has focused on targeting access to continuity models of care for women living in deprived areas and those from Black, Asian and minority ethnic groups [52, 53]. However, there is a significant knowledge gap around the mechanisms of continuity of care and specialist models. It is not known how and why some models of maternity care appear more effective than others, or if the positive outcomes reported in the literature are experienced by Black, Asian and minority ethnic women, and those with social risk factors. An expert panel in maternal and newborn health research, including service user representatives set global research priorities for the reduction of maternal and perinatal mortality, and preterm birth and stillbirths [54]. The top research priorities included ‘the evaluation of the effectiveness of midwifery care on access to family planning services, and rates of neonatal death, preterm birth and low birthweight’. Evaluating different models of care and identifying the impact of factors such as continuity of care and where antenatal care is received will help inform the organisation of future services for this ‘at risk’ population.

### Aim and objectives

#### Aim

To describe and compare maternal and neonatal clinical birth outcomes according to the model of maternity care women receive, and where their antenatal care is located.

#### Objectives

By comparing outcome data the analysis will explore whether standard maternity care, group practice or specialist models affect:

- Maternal and neonatal birth outcomes, the use of pharmacological analgesia and obstetric interventions.
- Women’s antenatal admissions to hospital and the length of their postnatal stay.

The analysis will also seek to identify:

- Sociodemographic characteristics associated with maternal and neonatal outcomes.
- Whether the location of antenatal care has an additional effect on maternal and neonatal outcomes?

## Methods

### Study design

The analysis reported in this paper is from a wider multi-site prospective observational study evaluating two UK based specialist models that provide maternity care to women with social risk factors. Demographic data for the first 500 women accessing maternity care in January 2019 at two large, inner-city maternity services were prospectively collected and anonymised. Pregnancy and birth outcome data were collected and analysed in August 2019 for women who had gone on to give birth at one of the two maternity services being evaluated. Exclusion criteria included those who experienced miscarriage (loss of pregnancy during the first 23 weeks), or who had not continued their antenatal care at the service. Three different models of maternity care with varying levels of continuity, and place of antenatal care were used as independent variables to explore their impact on pregnancy and birth outcomes. The research was approved by the London Brent Research Ethics Committee (HRA) REC Reference 18-LO-0701. This study is reported as per the STROBE checklist for observational studies [55].

### Setting

Two inner city National Health Service (NHS) maternity service providers in the UK that provide care to a multi-cultural, socioeconomically diverse population were purposively selected. As current policy and literature on improving maternal and neonatal health inequalities recommends relational continuity of care [40, 30, 56, 57], we selected providers that offered well-established specialist models of care aiming to provide continuity throughout the antenatal, intrapartum and postnatal period, as well as standard maternity care; and group practice to allow for comparisons between the three. Table 1 in S1 Appendix provides detailed definitions of the two service provider settings and the different models of care which women might experience at each:

### Data sources and variables

Outcome variables were collected based on the availability and comparability of compulsory data recorded within each service providers computerised data collection programmes ‘Cerner’ and ‘Badgernet’. To address potential sources of bias the data were collected and anonymised by clinicians outside of the research team.

Deprivation deciles, calculated using the 2019 English Indices of Deprivation [58] were grouped into four groups of sufficient numbers to enable comparisons between groups of similar numbers. These groups will be used throughout the findings chapters and are as follows:

1. Most deprived- 1^st^ and 2^nd^ deciles
2. 3^rd^ and 4^th^ deciles
3. 5^th^ and 6^th^ deciles
4. Least deprived- 7^th^, 8^th^, 9^th^ and 10^th^ deciles

Table 2 shows variables collected at each time point that will be presented in this paper. See Table 2 in S1 Appendix for definitions of variables.

**Table 2:** Outcome variables collected at two time points.

Outcome variable	1^st^ data collection- January 2019	2^nd^ data collection- August 2019
**Characteristics**		
Deprivation score	x	
Maternal age	x	
Ethnicity	x	
Parity	x	
Social risk factors (listed)		x
No. of social risk factors		x
Medical risk status at booking	x	
Medical risk status at onset of labour		x
**Service use**		
Reason if sample drop out		x
Model of care received		x
Place of birth		x
Neonatal unit admission		x
Length of postnatal stay		x
**Birth outcomes**		
Mode of birth		x
Induction of labour		x
Monitoring (CTG in labour)		x
Perineal trauma req suturing		x
Estimated blood loss		x
Analgesia		x
Obstetric emergency		x
Maternal death		x
**Neonatal Outcomes**		
Sex		x
Gestation at birth		x
Weight		x
Stillbirth/neonatal death		x
Apgar scores		x
Skin-to-skin		x
Feeding method		x
**Discharge information**		
Date discharged home		x
Social care involvement		x
Baby discharged home with parents/ LAC		x

### Sample size

A power calculation was based on a previous analysis of antenatal care utilisation in the UK [59] and research carried out on metrics for monitoring local inequalities in access to maternity care at the same service evaluated in this research [60]. This was due to the primary outcome of the full evaluation being access and engagement with maternity care. We calculated that with 250 women in each group (those receiving standard maternity care and those receiving group practice or a specialist model of care), we would have 90% power to detect a 15% difference in timely access (before 12 weeks’ gestation) to antenatal care between the different models of care with 500 anonymised birth records accessed at each trust. As the study was not primarily powered to detect differences in the maternal and neonatal birth outcomes analysed for this paper, a retrospective power analysis using previous literature on the relationship between premature birth and specialist maternity care was calculated [38, 61] demonstrating the sample size was underpowered to detect previously reported differences. Despite this, statistical significance was identified for numerous outcomes, highlighting the usefulness of this analysis.

**Fig 1:**
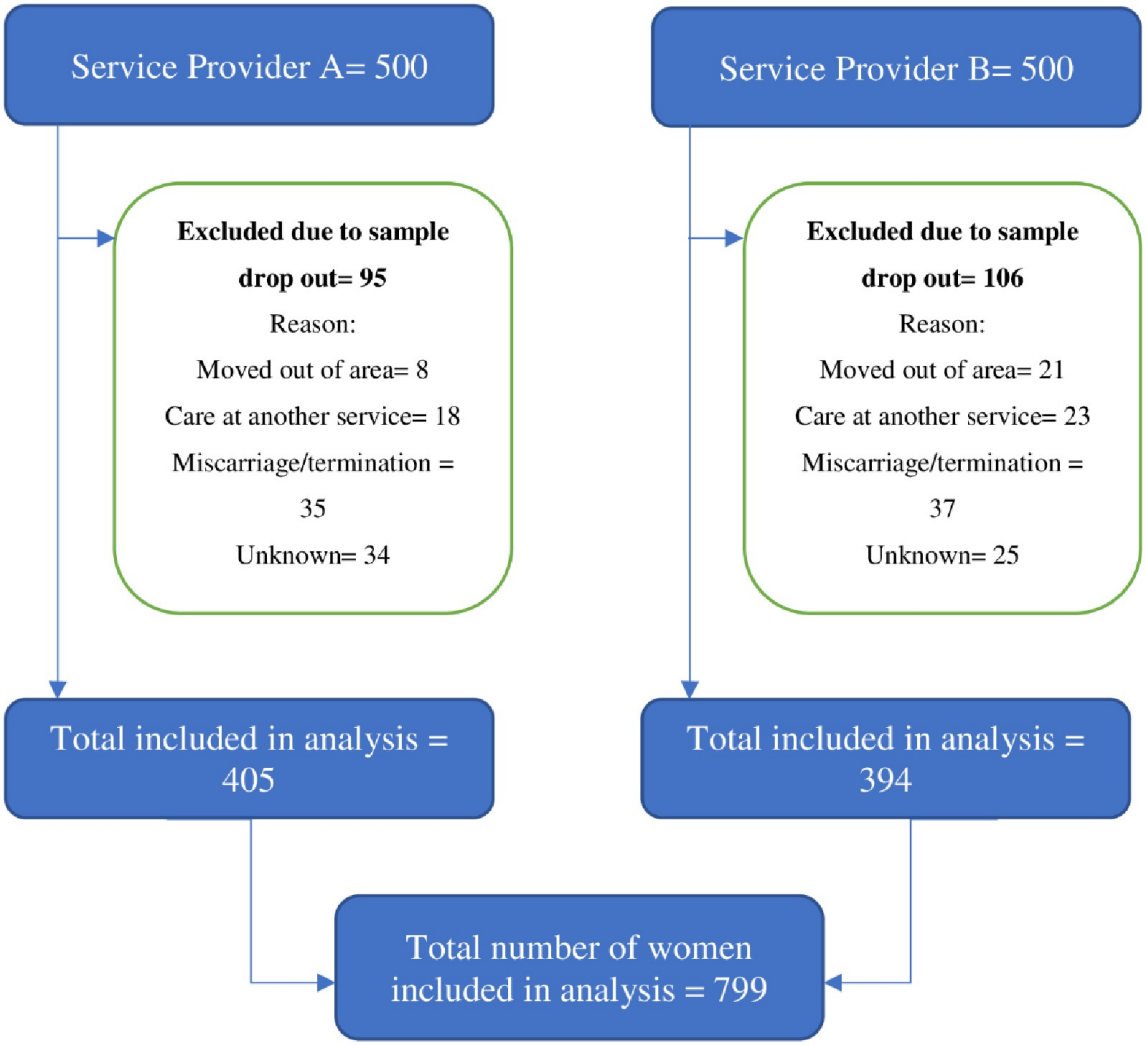
Data collection flowchart.

See Fig 1 for the data collection flowchart. Full pregnancy and birth data were collected and analysed from 799 women accessing care across the two service providers. Two hundred and one sets of birth outcome data were missing and were therefore excluded from the final analysis. Reasons for this missing data are reported in the findings.

### Statistical methods

The quantitative data were analysed using Stata 16.0. Firstly, women’s social risk factors, ethnicity, socioeconomic status and medical characteristics were described using descriptive statistics and stratified by the service provider attended to enable comparisons of differences in the samples between each service. It was decided to merge the two service providers outcome data for ease of interpreting the findings, with adjustment allowing for differences between the service providers. Variables were tested for bivariate association using chi-square tests and *t*-tests, for dichotomous and continuous variables, respectively. Chi-square analyses were also performed to test for associations between socio-economic position by deprivation (IMD) decile [58], as well as social and medical risk factors.

Three regression models were developed to identify the differences in the effect size for each: Model 1 adjusted for ethnicity, age, parity, deprivation score, social risk factors and medical risk status, Model 2 included model 1, plus adjustment for the service provider that women attended to consider differences in organisation guidelines, processes and culture and Model 3 included model 2, however, the place of antenatal care (hospital versus community-based antenatal care) was treated as the independent variable. This structured model allowed us to explore the association between maternal and neonatal outcomes depending on the model of care received, whilst accounting for interactions between independent variables to predict the dependent variable. A subgroup analysis of statistically significant findings was also conducted for those women who are at highest risk of poor birth outcomes. Where pregnancy and birth outcomes are presented risk ratios and confidence intervals are used to demonstrate statistical significance as well as the direction and strength of the effect [62].

## Results

The section below describes the characteristics of the women in the quantitative sample. The women who were excluded due to drop out are presented first to explore differences between the two service providers and deprivation groups. P values are presented to show statistical significance between the characteristics of women accessing maternity care at both providers.

### Characteristics of women with missing outcome data

Of the first 1000 women who had an appointment to book for maternity care in 2019, 201 did not go on to give birth at the service and are not included in the quantitative data analysis that follows. The total numbers of women with missing outcome data at each hospital did not differ significantly, allowing the data from both services to be pooled without having to adjust for missing data. No significant difference was found in the reason for missing data when analysed according to women’s deprivation scores.—See Tables 1 and 2 in S2 Appendix. The small sample size here should be kept in mind; an apparent trend may have become significant with a larger sample size, reflecting the literature demonstrating a social gradient for both spontaneous miscarriage and termination of pregnancy rates [13, 14, 21–23].

### Demographics of women included in the quantitative data analysis

The section below describes the demographics of the 799 women who went on to receive antenatal care and give birth at one of the two service providers. Table 3 shows that more women at service A were recorded as ‘white British’, and more women at service B were recorded as ‘white other’. Ethnicity was also more likely to be recorded as ‘unknown’ at service A. Women at service B were more likely to have at least one social risk factor recorded, have common mental health issues, drug and/or alcohol abuse, financial and/or housing issues, be non-English speaking, unsupported, and have disclosed female genital mutilation (FGM). Women at service B were also significantly more likely to have high medical risk status at the onset of labour. Similar numbers of women experienced standard care and private (Non-NHS) care at both service providers. However, more women at service B received the group practice model, and more women at service A received specialist models of care. More women at service B experienced standard care in the hospital setting whereas more women at service A experience standard care based in the community setting. Women receiving private care were not included in the analysis as private care is not a realistic option for women with low socioeconomic status, and numbers were too small to gain generalisable learning. The differences reported here informed the modelling structure that adjusted for women’s demographics and risk factors.

**Table 3:** Women’s demographics at each service provider.

Demographic variable	Service A n(%)	Service B n(%)	TOTAL n(%)	X^2^ p value
	Total data = 405	Total data = 394	Total data = 799	
**Ethnicity**				**Pr 0.000**
Asian	37(9)	53(13)	90(11)	
Black African	31(8)	46(12)	77(10)	
Black Caribbean	23(6)	16(4)	39(5)	
Black other	8(2)	14(4)	22(3)	
Mixed	12(3)	7(2)	19(2)	
White British	98(24)	58(15)	156(20)	
White other	80(20)	139(36)	219(27)	
Unknown	116(29)	61(15)	177(22)	
**Age**				Pr 0.356
≤20	6(1)	4(1)	10(1)	
21–24 years	19(5)	32(8)	51(6)	
25–29 years	63(16)	56(14)	119(15)	
30–34 years	134(33)	125(32)	259(32)	
≥35 years	183(45)	177(45)	360(45)	
**IMD Quintile (2019)**				**Pr 0.035**
Most deprived (1^st^ +2^nd^ deciles)	92(23)	114(29)	206(26)	
3^rd^ and ^4th^ deciles	160(40)	126(32)	286(36)	
5^th^ and 6^th^ deciles	72(18)	86(22)	158(20)	
Least deprived (7^th^, 8^th^, 9^th^ +10^th^ deciles)	81(20)	68(17)	149(19)	
**Social Risk Factor**				
Domestic abuse	23(6)	17(4)	40(5)	Pr 0.377
Common mental health	4(1)	34(9)	38(5)	**Pr 0.000**
Severe mental health	2(<1)	8(2)	10(1)	Pr 0.051
Non-English speaking	16(4)	48(13)	64(8)	**Pr 0.000**
Social care involvement	27(7)	29(7)	56(7)	Pr 0.701
Drug/alcohol abuse	1(<1)	10(3)	11(1)	**Pr 0.005**
Unsupported/single	1(<1)	11(3)	12(2)	**Pr 0.003**
Financial/housing	15(4)	31(8)	46(6)	**Pr 0.012**
Learning disability	6(2)	5(1)	11(1)	Pr 0.797
Sexual abuse/trafficked	4(2)	5(1)	9(1)	Pr 0.677
AS/Refugee	8(2)	7(2)	15(2)	Pr 0.836
FGM	0	11(3)	11(1)	**Pr 0.001**
No recourse to public funds	6(1)	0	6(1)	**Pr 0.015**
**No of social risk factors**				**Pr 0.003**
None	337(83)	279(70)	616(77)	
1	43(11)	61(15)	104(13)	
2	13(3)	26(7)	39(5)	
3	6(1)	15(4)	21(3)	
4	5(1)	9(2)	14(2)	
≥5	1(<1)	4(1)	5(1)	
**Name of model of care**				**Pr 0.000**
Standard Care	256(63)	213(54)	469(59)	
Group Practice	77(19)	144(37)	221(28)	
Specialist	59(15)	21(5)	80(10)	
**Private Care**	13(3)	16 (4)	29(4)	
**Place of model of antenatal care**				**Pr 0.000**
Standard model in hospital	100(25)	212(54)	312(39)	
Standard model in community	156(40)	1(0)	157(20)	
Group practice in community	40(10)	94(24)	134(17)	
Group practice in hospital	37(9)	50(13)	87(11)	
Specialist model in community	59(15)	2(1)	61(8)	
Specialist model in hospital	0	19(5)	19(2)	
Private Care	13(3)	16(4)	29(4)	
By place of antenatal care only*				**Pr 0.000**
Hospital based	137(35)	281(74)	418(54)	
Community based	255(65)	97(26)	352(46)	

*Excludes private care.

Significant differences were found in the care received by women depending on their deprivation score- See Table 3 in S2 Appendix. Women in the most deprived deciles were significantly more likely to receive a specialist model of care, and women in the least deprived deciles were less likely to receive community based antenatal care than hospital based antenatal care. A statistically significant relationship was also found between deprivation score and the number of social risk factors recorded, reflecting the literature showing the lower a woman’s socio-economic status, the more likely she is to be experiencing one of more social risk factors [63–67].

## Outcome data

### Maternal birth outcomes

#### Analysis 1- model of care

The data presented in Table 4 tests the hypothesis that maternal birth outcomes will vary according to model of care. No significant relationship was found between the model of care received and women’s birth outcomes, including mode of birth, blood loss, perineal trauma requiring suturing, and obstetric emergencies, after adjusting for women’s characteristics and service differences. When adjusting for women’s characteristics- See Table 3 in S2 Appendix, null parity was found to be a significant predictor of increased emergency caesarean section (RR4.96 CI 3.09–7.94) and instrumental delivery (RR 8.06 CI 4.71–13.79). Women at high medical risk at booking (RR 5.52 CI 2.20–13.83) and at the onset of labour (RR 2.66 CI 1.47–4.81) were more likely to have an elective or emergency caesarean, and instrumental delivery compared to women with low medical risk. Primiparous women were more likely to have a postpartum haemorrhage (PPH) (RR 3.23 CI 2.32–4.50), perineal trauma requiring suturing (RR 2.30 CI 1.67–3.17) and experience an obstetric emergency (RR1.94 CI 1.34–2.79). Women with high medical risk status at the onset of labour were also more likely to have a postpartum haemorrhage (PPH) (RR 1.84 CI 1.26–2.69), other obstetric emergency (RR1.77 CI 1.15–2.75), and less likely to have perineal trauma requiring suturing (RR 0.47 CI 0.32–0.69). A significant relationship was found between women with any social risk factor and massive obstetric haemorrhage (MOH) (RR 1.99 CI 1.03–3.83). Maternal death was not included in the analysis as numbers were too small to detect a relationship (n = 1).

**Table 4:** Maternal birth outcomes and model of care.

Birth outcome	Model of Care	No. of women (%)	Unadjusted RR	Model 1	Model 2	Model 3
			(95% CI)	Adjusted RR (95% CI) *	Adjusted RR(95% CI) **	Adjusted RR(95% CI) ***
Spontaneous vaginal birth	Standard	209(54)	Ref	Ref	Ref	Ref
	Group	132(34)	Ref	Ref	Ref	Ref
	Specialist	44(12)	Ref	Ref	Ref	Ref
Instrumental delivery	Standard	82(67)	1.91(0.89–4.10)	1.75(0.75–4.09)	1.59(0.66–3.81)	1.60(0.67–3.83)
	Group	32(26)	1.18(0.40–1.45)	1.23(0.49–3.04)	1.20(0.48–2.98)	1.18(0.46–2.99)
	Specialist	9(7)	Ref	Ref	Ref	Ref
Emergency caesarean section	Standard	91(59)	1.00(0.55–1.81)	0.97 (0.48–1.95)	0.91(0.44–1.86)	0.91(0.44–1.88)
	Group	44(29)	0.77(0.40–1.45)	0.33 (0.33–1.46)	0.68(0.32–1.44)	0.65(0.30–1.40)
	Specialist	19(12)	Ref	Ref	Ref	Ref
Elective caesarean section	Standard	87(81)	**2.28(1.03–5.06)**	2.28(0.94–5.51)	1.91(0.77–4.72)	2.00(0.80–4.99)
	Group	13(12)	0.54(0.21–1.39)	0.47(0.16–1.33)	0.43(0.15–1.24)	0.36(0.12–1.05)
	Specialist	8(7)	Ref	Ref	Ref	Ref
Blood loss>500mls (PPH)	Standard	249(64)	1.25(0.77–2.01)	1.07(0.63–1.82)	1.02(0.59–1.76)	1.02(0.59–1.76)
	Group	102(26)	0.94 (0.56–1.58)	0.91(0.51–1.61)	0.90(0.51–1.59)	0.76(0.42–1.37)
	Specialist	38(10)	Ref	Ref	Ref	Ref
Blood loss> 1000mls (MOH)	Standard	40(65)	0.73(0.34–1.58)	0.88(0.38–2.03)	1.00(0.42–2.36)	0.99(0.41–2.34)
	Group	13(21)	0.49(0.20–1.20)	0.57(0.22–1.48)	0.59(0.22–1.54)	0.69(0.26–1.83)
	Specialist	9(15)	Ref	Ref	Ref	Ref
Perineal trauma req suturing	Standard	199(60)	1.22 (0.75–2.00)	1.17(0.68–2.02)	1.11(0.63–1.95)	1.11(0.63–1.94)
	Group	101(31)	1.40 (0.83–2.36)	1.47(0.82–2.64)	1.45(0.81–2.61)	1.38(0.76–2.51)
	Specialist	30(9)	Ref	Ref	Ref	Ref
Obstetric emergency	Standard	119(63)	1.17(0.66–2.07)	1.14(0.62–2.10)	1.20(0.64–2.25)	1.21(0.65–2.25)
	Group	53(28)	1.09(0.59–2.02)	1.19(0.62–2.29)	1.22(0.63–2.34)	1.29(0.66–2.51)
	Specialist	18(9)	Ref	Ref	Ref	Ref

* Model 1: Adjusted for demographics ethnicity, age, parity, IMD score, any social risk factor and medical risk factors at booking and onset of labour.

** Model 2: Model 1 + Adjustment for place of antenatal care (community or hospital).

*** Model 3: Model 2 + Adjustment for service provider attended (A or B).

#### Analysis 2- place of antenatal care

A second analysis was run on the impact of place of antenatal care on birth outcomes. Table 5 shows that, after adjusting for potential confounders, there was no significant relationship between the place of antenatal care and maternal birth outcomes.

**Table 5:** Maternal birth outcomes in relation to the place of antenatal care.

Birth outcome	Place of antenatal care	Number of women n(%)	Unadjusted RR	Model 1	Model 2	Model 3
			(95% CI)	Adjusted RR (95% CI) *	Adjusted RR (95% CI) **	Adjusted RR (95% CI) ***
Spontaneous vaginal birth	Hospital	187(49)	Ref	Ref	Ref	Ref
	Community	198(51)	Ref	Ref	Ref	Ref
Instrumental delivery	Hospital	69(56)	1.34(0.89–2.02)	1.43(0.90–2.26)	1.27(0.78–2.07)	1.24(0.74–2.10)
	Community	54(44)	Ref	Ref	Ref	Ref
Emergency caesarean	Hospital	88(57)	1.38(0.95–2.02)	1.27(0.82–1.97)	1.19(0.75–1.89)	1.12(0.68–1.83)
	Community	66(43)	Ref	Ref	Ref	Ref
Elective caesarean	Hospital	74(69)	**2.26(1.43–3.55)**	**2.10(1.26–3.48)**	1.61(0.92–2.80)	1.06(0.56–2.01)
	Community	34(31)	Ref	Ref	Ref	Ref
PPH (Blood loss>500mls)	Hospital	227(58)	**1.37(1.03–1.82)**	1.16(0.84–1.60)	1.11(0.79–1.55)	0.92(0.64–1.33)
	Community	162(42)	Ref	Ref	Ref	Ref
MOH (Blood loss> 1L)	Hospital	30(52)	0.77(0.46–1.30)	0.76 (0.43–1.34)	0.71(0.39–1.30)	0.88(0.46–1.69)
	Community	30(48)	Ref	Ref	Ref	Ref
Perineal trauma req suturing	Hospital	155(47)	0.92(0.69–1.23)	1.08(0.78–1.49)	1.15(0.82–1.61)	1.08(0.75–1.56)
	Community	175(53)	Ref	Ref	Ref	Ref
Obstetric emergency	Hospital	89(47)	0.91(0.66–1.27)	0.89(0.62–1.27)	0.85(0.58–1.24)	0.91(0.61–1.37)
	Community	101(53)	Ref	Ref	Ref	Ref

* Model 1: Adjustment for demographics ethnicity, age, parity, IMD score, social risk and medical risk factors at booking and onset of labour.

** Model 2: Model 1 + adjustment for model of care.

*** Model 3: Model 2 + adjustment for service provider attended.

### Analgesia in labour and obstetric interventions

#### Analysis 1- model of care

Table 6 shows that the only statistically significant relationship with model of care across all unadjusted and adjusted models was the use of water in labour. Women receiving the specialist model of care were most likely to use water to relive pain during labour, with those receiving standard care being least likely (RR 0.11 CI 0.02–0.62). When adjusting for women’s characteristics those with high medical risk status at onset of labour (RR4.57 CI 2.97–7.503) and those over 34 years old (RR 5.85 CI 1.39–24.55) were significantly more likely to have an epidural. Primiparous women were most likely to have an epidural (RR 0.55 CI 0.37–0.82) and opioid analgesia (RR 4.81 CI 1.19–19.35). Differences seen in the number of women having a CTG in labour was largely driven by primiparous women (RR1.68 CI 1.06–2.64) and those with high medical risk status at the onset of labour (RR3.06 CI 1.94–4.83).

**Table 6:** Use of analgesia in labour and obstetric interventions in relation to the model of care received.

Analgesia in labour/Intervention	Model of Care	Number of women (%)	Unadjusted RR	Model 1	Model 2	Model 3
			(95% CI)	Adjusted RR (95% CI) *	Adjusted RR (95% CI) **	Adjusted RR (95% CI) ***
Epidural/CSE/GA	Standard	306(64)	1.30(0.80–2.11)	0.99(0.56–1.73)	1.01(0.57–1.80)	1.01(0.57–1.80)
	Group	123(26)	0.89(0.53–1.49)	0.72(0.39–1.32)	0.73(0.40–1.33)	0.71(0.38–1.31)
	Specialist	47(10)	Ref	Ref	Ref	Ref
Opioid analgesia	Standard	9(60)	0.76(0.16–3.59)	0.55(0.10–2.89)	0.54(0.09–3.17)	0.51(0.87–3.05)
	Group	4(27)	0.71(0.12–4.00)	0.56(0.08–3.53)	0.56(0.08–3.53)	0.29(0.03–2.51)
	Specialist	2(13)	Ref	Ref	Ref	Ref
No analgesia or Entonox	Standard	90(53)	0.62(0.36–1.07)	0.70(0.38–1.29)	0.74(0.30–1.38)	0.73(0.39–1.37)
	Group	57(34)	0.96(0.51–1.62)	1.00(0.52–1.90)	1.01(0.53–1.93)	1.15(0.59–2.22)
	Specialist	22(13)	Ref	Ref	Ref	Ref
Water in labour	Standard	3(23)	**0.09(0.02–0.41)**	**0.10(0.02–0.53)**	**0.14(0.02–0.72)**	**0.11(0.02–0.62)**
	Group	5(38)	0.34(0.09–1.23)	0.48(0.10–2.22)	0.50(0.10–2.31)	0.65(0.14–3.06)
	Specialist	5(38)	Ref	Ref	Ref	Ref
CTG in labour	Standard	168(60)	**2.28 (1.27–4.07)**	1.17(0.86–3.39)	0.96(0.45–2.02)	0.92(0.38–2.19)
	Group	97(34)	**3.15(1.71–5.80)**	**2.69(1.31–5.48)**	**2.84 (1.34–6.01)**	0.80(0.32–2.01)
	Specialist	16(6)	Ref	Ref	Ref	Ref
Induction of labour	Standard	203(60)	0.89(0.55–1.43)	0.89(0.52–1.52)	1.10(0.63–1.91)	1.10(0.63–1.91)
	Group	97(29)	0.90(0.54–1.51)	0.85(0.48–1.51)	0.90(0.50–1.61)	1.01(0.56–1.83)
	Specialist	37(11)	Ref	Ref	Ref	Ref

* Model 1: Adjusted for demographics ethnicity, age, parity, IMD score, any social and medical risk factors at booking and onset of labour.

** Model 2: Model 1 + Adjustment for place of antenatal care (community or hospital).

*** Model 3: Model 2 + Adjustment for service provider attended (A or B).

#### Analysis 2- place of antenatal care

Table 7 shows no significant relationship between the place of antenatal care and use of analgesia. However a significant relationship was found for women receiving antenatal care in the hospital being less likely to experience an induction of labour (RR0.65 CI 0.45–0.95). The differences in the use of water for pain relief in labour were driven by the significant relationship with the model of care received.

**Table 7:** Use of analgesia in labour and obstetric intervention in relation to the place of antenatal care.

Analgesia/Intervention	Place of antenatal care	Number of women (%)	Unadjusted RR	Model 1	Model 2	Model 3
			(95% CI)	Adjusted RR (95% CI) *	Adjusted RR (95% CI) **	Adjusted RR (95% CI) ***
Epidural/CSE/GA	Hospital	271(57)	1.33(0.99–1.78)	1.00(0.71–1.41)	0.93(0.65–1.33)	0.90(0.61–1.32)
	Community	203(43)	Ref	Ref	Ref	Ref
Opioid analgesia	Hospital	8(53)	0.96(0.34–2.68)	0.93(0.31–2.80)	1.01(0.29–3.52)	0.59(0.12–2.93)
	Community	7(47)	Ref	Ref	Ref	Ref
No analgesia or Entonox	Hospital	76(45)	**0.62(0.43–0.87)**	0.79(0.54–1.16)	0.87(0.58–1.28)	1.01(0.66–1.54)
	Community	93(55)	Ref	Ref	Ref	Ref
Water in labour	Hospital	3(23)	**0.24(0.06–0.90)**	0.28(0.06–1.15)	0.42(0.09–1.94)	0.70(0.14–3.52)
	Community	10(77)	Ref	Ref	Ref	Ref
CTG in labour	Hospital	73(26)	**3.88(2.81–5.36)**	**2.83(1.95–4.09)**	**4.18(2.70–6.49)**	1.08(0.61–1.92)
	Community	210(74)	Ref	Ref	Ref	Ref
Induction of labour	Hospital	171(51)	0.76(0.57–1.01)	**0.60(0.43–0.84)**	**0.57(0.40–0.80)**	**0.65(0.45–0.95)**
	Community	166(49)	Ref	Ref	Ref	Ref

* Model 1: Adjustment for demographics ethnicity, age, parity, IMD score, social risk and medical risk factors at booking and onset of labour.

** Model 2: Model 1 + adjustment for model of care.

*** Model 3: Model 2 + adjustment for service provider attended.

### Place of birth

#### Analysis 1- model of care

Table 8 shows that overall, there was no significant difference between the model of care and place of birth. However, women attending service provider B were significantly more likely to give birth on the labour ward (RR 4.15 CI 2.46–7.00). See Table 19 in S2 Appendix for fully adjusted outcome tables.

**Table 8:** Place of birth in relation to the model of care received.

Place of birth	Model of Care	Number of women (%)	Unadjusted RR	Model 1	Model 2	Model 3
			(95% CI)	Adjusted RR (95% CI) *	Adjusted RR (95% CI) **	Adjusted RR (95% CI) ***
Birth Centre/midwife led setting	Standard	119(57)	Ref	Ref	Ref	Ref
	Group	61(29)	Ref	Ref	Ref	Ref
	Specialist	28(14)	Ref	Ref	Ref	Ref
Labour ward/obstetric led setting	Standard	343(62)	**1.64(0.99–2.74)**	1.41(0.77–2.58)	1.08(0.58–2.02)	1.11(0.58–2.12)
	Group	157(29)	1.47(0.84–2.55)	1.29(0.67–2.49)	1.19(0.61–2.31)	0.76(0.38–1.53)
	Specialist	49(9)	Ref	Ref	Ref	Ref
Home	Standard	2(25)	**0.15(0.02–0.98)**	**0.13(0.01–0.99)**	0.16(0.01–1.41)	0.16(0.01–1.45)
	Group	3(38)	0.45(0.08–2.42)	0.39(0.05–2.67)	0.43(0.06–2.99)	0.34(0.03–2.99)
	Specialist	3(38)	Ref	Ref	Ref	Ref

* Model 1: Adjusted for demographics ethnicity, age, parity, IMD score, any social and medical risk factors at booking and onset of labour.

** Model 2: Model 1 + Adjustment for place of antenatal care (community or hospital).

*** Model 3: Model 2 + Adjustment for service provider attended (A or B).

#### Analysis 2- place of antenatal care

Table 9 shows no significant relationship between place of antenatal care and place of birth once the model adjusted for the service attended.

**Table 9:** Place of birth in relation to place of antenatal care.

Place of birth	Place of antenatal care	No of women (%)	Unadjusted RR	Model 1	Model 2	Model 3
			(95% CI)	Adjusted RR (95% CI) *	Adjusted RR (95% CI) **	Adjusted RR (95% CI) ***
Birth Centre/midwife led setting	Hospital	75(36)	Ref	Ref	Ref	Ref
	Community	133(64)	Ref	Ref	Ref	Ref
Labour ward/obstetric led setting	Hospital	340(62)	**2.86(2.05–3.99)**	**2.06(1.40–3.01)**	**2.06(1.38–3.07)**	1.31(0.85–2.02)
	Community	209(38)	Ref	Ref	Ref	Ref
Home	Hospital	2(25)	0.59(0.11–3.00)	0.40(0.06–2.55)	0.74(0.09–5.60)	0.58(0.07–4.70)
	Community	6(75)	Ref	Ref	Ref	Ref

* Model 1: Adjustment for demographics ethnicity, age, parity, IMD score, social risk and medical risk factors at booking and onset of labour.

** Model 2: Model 1 + adjustment for model of care.

*** Model 3: Model 2 + adjustment for service provider attended.

### Neonatal outcomes

#### Analysis 1- model of care

Table 10 shows no significant relationship between the model of care received and neonatal outcomes. When adjusting for women’s characteristics- see Table 21 in S2 Appendix, neonates of primiparous women were significantly more likely to have low birth weight (RR1.85 CI 1.07–3.20), as were neonates of women with high medical risk status as the onset of labour (RR 2.83 CI 1.4305.61). Neonates of women with any social risk factor (RR 2.52 CI 1.02–6.17), and Black Caribbean women (RR11.86 CI 1.23–114.3) were more likely to have a low Apgar score (<8 at 5 minutes), although CI’s were wide. Neonatal unit admissions were more likely for Black African women (RR 3.99 CI 1.37–11.64) and those with high medical risk status at the onset of labour (RR 4.06 CI 2.10–7.84). Neonatal death and stillbirth was not included in the analysis as numbers in each model of care were too small to detect a relationship (n = 8).

**Table 10:** Neonatal outcomes in relation to the model of care received.

Neonatal outcome	Model of Care	No. of women (%)	Unadjusted RR	Model 1	Model 2	Model 3
			(95% CI)	Adjusted RR (95% CI) *	Adjusted RR (95% CI) **	Adjusted RR (95% CI) ***
Gestation <37 weeks at birth	Standard	52(61)	1.12(0.51–2.46)	1.11(0.47–2.62)	0.81(0.33–1.99)	0.80(0.33–1.98)
	Group	25(29)	1.14(0.49–2.66)	1.08(0.43–2.68)	0.96(0.38–2.43)	0.98(0.38–2.50)
	Specialist	8(10)	Ref	Ref	Ref	Ref
Birthweight <2500g***	Standard	45(63)	1.59(0.61–4.14)	1.60(0.57–4.45)	1.16(0.40–3.36)	1.16(0.40–3.36)
	Group	21(30)	1.57(0.57–4.32)	1.49(0.50–4.36)	1.24(0.41–3.73)	1.30(0.43–3.93)
	Specialist	5(7)	Ref	Ref	Ref	Ref
Apgar <8 at 5 minutes	Standard	19(59)	0.80(0.26–2.42)	1.44(0.40–5.23)	1.49(0.40–5.46)	1.46(0.39–5.37)
	Group	9(28)	0.80(0.24–2.69)	1.20(0.30–4.81)	1.22(0.30–4.84)	1.42(0.35–5.71)
	Specialist	4(13)	Ref	Ref	Ref	Ref
Neonatal unit admission	Standard	50(61)	1.78(0.69–4.63)	1.67(0.60–4.69)	1.31(0.45–3.80)	1.31(0.45–3.81)
	Group	27(33)	2.08(0.77–5.62)	1.77(0.60–5.22)	1.58(0.53–4.71)	1.59(0.53–4.80)
	Specialist	5(6)	Ref	Ref	Ref	Ref

* Model 1: Adjusted for demographics ethnicity, age, parity, IMD score, any social and medical risk factors at booking and onset of labour.

** Model 2: Model 1 + Adjustment for place of antenatal care (community or hospital).

*** Model 3: Model 2 + Adjustment for service provider attended (A or B).

#### Analysis 2- place of antenatal care

Table 11 shows that women receiving antenatal care in the hospital were significantly more likely to have a preterm birth (RR 2.38 CI 1.32–4.27) and neonatal low birth weight (RR 2.31 CI 1.24–4.32) than those receiving antenatal care in the community setting. These relationships were statistically significant across all models after adjusting for women’s characteristics, including their medical risk status, model of care received, and service provider attended. Although no relationship was found between the place of antenatal care and stillbirth or neonatal death, the adjusted analysis presented in Table 22 in S2 Appendix highlight the significance for women with any social risk factor being more likely to have a stillbirth or neonatal death (RR 6.82 CI 1.10–42.15).

**Table 11:** Neonatal outcomes in relation to the place of antenatal care.

Neonatal outcome	Place of antenatal Care	No. of women (%)	Unadjusted RR	Model 1	Model 2	Model 3
			(95% CI)	Adjusted RR (95% CI) *	Adjusted RR (95% CI) **	Adjusted RR (95% CI) ***
Gestation <37 at birth	Hospital	62(73)	**2.45(1.48–4.05)**	**2.18(1.28–3.72)**	**2.26(1.29–3.95)**	**2.38(1.32–4.27)**
	Community	23(27)	Ref	Ref	Ref	Ref
Birthweight <2500g*	Hospital	51(72)	**2.26(1.31–3.87)**	**2.20(1.23–3.92)**	**2.15(1.18–3.92)**	**2.31(1.24–4.32)**
	Community	20(28)	Ref	Ref	Ref	Ref
Apgar <8 at 5 minutes	Hospital	17(53)	0.89(0.43–1.83)	0.91(0.42–2.01)	0.82(0.36–1.85)	1.25(0.51–3.08)
	Community	15(47)	Ref	Ref	Ref	Ref
NNU admission	Hospital	57(70)	**1.98(1.20–3.26)**	**1.77(1.04–3.02)**	1.72(0.99–2.99)	1.74(0.97–3.11)
	Community	25(30)	Ref	Ref	Ref	Ref
Neonatal death	Hospital	4(50)	0.84(0.20–3.39)	0.65(0.14–2.90)	0.60(0.12–2.99)	0.98(0.20–4.72)
	Community	4(50)	Ref	Ref	Ref	Ref

* Model 1: Adjusted for demographics: ethnicity, age, parity, IMD score, social risk and medical risk factors at booking and onset of labour.

** Model 2: Model 1 plus adjusted for model of care.

*** Model 3: Model 2 plus Adjusted for service provider attended.

### Infant care

#### Analysis 1- model of care

Table 12 shows women were much less likely to have had skin-to-skin contact recorded if they received standard maternity care (RR 0.34 CI 0.14–0.80) and group practice care (RR 0.31 CI 0.13–0.74) compared to those receiving the specialist model. Other women least likely to have had skin-to-skin contact with their infants were Black Caribbean women (RR 0.40 CI 0.16–1.00), those with any social risk factor (RR0.59 CI0.38–0.92), women with high medical risk status (RR 0.32 CI 0.21–0.50) and those attending service provider B (RR 0.39 CI 0.22–0.68). No significant relationship was found between model of care and method of infant feeding at discharge from hospital. When the model adjusted for women’s characteristics, women with high medical risk status were significantly more likely to be feed their infants artificially (RR2.80 CI 1.33–5.89) or mixed feed (RR 1.78 CI 1.13–2.81). Black Caribbean women were more likely to artificially feed (RR 12.67 CI 1.34–11.8) and those in the Black ‘other’ ethnic category were more likely to mixed feed (RR 4.26 CI 1.50–12.08).

**Table 12:** Feeding method and skin-to-skin in relation to model of care.

Feeding method and skin-to-skin	Model of Care	Number of women (%)	Unadjusted RR	Model 1	Model 2	Model 3
			(95% CI)	Adjusted RR (95% CI) *	Adjusted RR (95% CI) **	Adjusted RR (95% CI) ***
Breastfeeding at discharge	Standard	309(61)	Ref	Ref	Ref	Ref
	Group	147(29)	Ref	Ref	Ref	Ref
	Specialist	52(10)	Ref	Ref	Ref	Ref
Artificially feeding at discharge	Standard	32(55)	1.07(0.40–2.89)	1.89(0.63–5.63)	1.69(0.55–5.17)	1.69(0.55–5.14)
	Group	21(36)	1.48(0.53–4.14)	2.10(0.67–6.50)	2.03(0.65–6.35)	2.47(0.78–7.78)
	Specialist	5(9)	Ref	Ref	Ref	Ref
Mixed Feeding at discharge	Standard	125(63)	0.91(0.53–1.55)	1.10(0.60–1.94)	1.12(0.61–2.05)	1.12(0.61–2.04)
	Group	59(25)	0.75(0.41–1.35)	0.89(0.47–1.69)	0.91(0.48–1.72)	1.16(0.60–2.24)
	Specialist	23(12)	Ref	Ref	Ref	Ref
Skin-to-skin	Standard	348(61)	**0.41(0.20–0.82)**	**0.28(0.12–0.63)**	**0.35(0.15–0.80)**	**0.34(0.14–0.80)**
	Group	156(27)	**0.34(0.16–0.70)**	**0.25(0.10–0.58)**	**0.26(0.11–0.61)**	**0.31(0.13–0.74)**
	Specialist	70(12)	Ref	Ref	Ref	Ref

* Model 1: Adjusted for demographics ethnicity, age, parity, IMD score, any social and medical risk factors at booking and onset of labour.

** Model 2: Model 1 + Adjustment for place of antenatal care (community or hospital).

*** Model 3: Model 2 + Adjustment for service provider attended (A or B).

#### Analysis 2- place of antenatal care

Table 13 shows that there was no relationship between infant care outcomes and place of antenatal care. For skin-to-skin contact after birth there appeared to be a difference, but when the model adjusted for provider we see that the relationship was driven by women attending service B being less likely to have had skin to skin contact (RR 0.39 CI 0.22–0.68).

**Table 13:** Feeding method and skin-to-skin contact in relation to place of antenatal care.

Feeding method and skin-to-skin	Place of antenatal care	Number of women (%)	Unadjusted RR	Model 1	Model 2	Model 3
			(95% CI)	Adjusted RR (95% CI) *	Adjusted RR (95% CI) **	Adjusted RR (95% CI) ***
Breastfeeding at discharge	Hospital	276(53)	Ref	Ref	Ref	Ref
	Community	232(46)	Ref	Ref	Ref	Ref
Artificially feeding	Hospital	36(62)	1.29(0.73–2.27)	1.45(0.79–2.65)	1.37(0.73–2.57)	1.85(0.94–3.65)
	Community	22(38)	Ref	Ref	Ref	Ref
Mixed Feeding	Hospital	104(53)	0.93(0.67–1.30)	0.94(0.65–1.35)	0.90(0.61–1.31)	1.23(0.82–1.86)
	Community	93(47)	Ref	Ref	Ref	Ref
Skin-to-skin	Hospital	281(49)	**0.42(0.29–0.59)**	**0.52(0.35–0.76)**	**0.53(0.35–0.80)**	0.69(0.44–1.07)
	Community	293(51)	Ref	Ref	Ref	Ref

* Model 1: Adjusted for demographics ethnicity, age, parity, IMD score, social risk and medical risk factors.

** Model 2: Model 1 plus adjusted for model of care.

*** Model 3: Model 2 plus Adjusted for service provider attended.

### Service use

#### Analysis 1- model of care

Table 14 shows no significant relationship between the model of care received and the number of antenatal admissions to hospital, or the length of the postnatal stay. However, Black Caribbean (RR 2.86 CI 1.11–7.38) and ‘Black other’ women (RR 3.59 CI 1.15–11.17) were more likely to have one or more antenatal admissions- see Table 25 in S2 Appendix. Once adjustment was made for provider, women at service B (RR 3.46 CI 1.84–6.50) and those with high medical risk (2.64 CI 1.67–4.18) were more likely to have one or more antenatal admissions, and to stay in hospital after giving birth for 4 or more days (RR 3.91 CI 2.18–7.00).

**Table 14:** Women’s service use in relation to the model of care received.

Service use	Model of care	Number of women (%)	Unadjusted RR	Model 1	Model 2	Model 3
			(95% CI)	Adjusted RR (95% CI) *	Adjusted RR (95% CI) **	Adjusted RR (95% CI) ***
1 or more antenatal admissions	Standard	90(60)	1.02(0.56–1.88)	1.04(0.52–2.07)	0.90(0.44–1.84)	0.89(0.43–1.86)
	Group	46(30)	1.13(0.59–2.17)	1.10(0.53–2.29)	1.07(0.51–2.25)	0.81(0.37–1.76)
	Specialist	15(10)	Ref	Ref	Ref	Ref
Length of postnatal stay:						
0–1 day	Standard	227(60)	Ref	Ref	Ref	Ref
	Group	116(30)	Ref	Ref	Ref	Ref
	Specialist	36(10)	Ref	Ref	Ref	Ref
2 days	Standard	118(66)	0.93(0.51–1.68)	0.88(0.45–1.70)	0.85(0.43–1.67)	0.85(0.43–1.68)
	Group	42(23)	0.65(0.34–1.24)	0.60(0.29–1.24)	0.60(0.29–1.23)	0.52(0.25–1.11)
	Specialist	20(11)	Ref	Ref	Ref	Ref
3 days	Standard	54(57)	0.85(0.40–1.83)	0.84(0.36–1.93)	0.90(0.38–2.13)	0.91(0.38–2.14)
	Group	30(32)	0.93(0.41–2.08)	0.83(0.34–2.00)	0.85(0.35–2.06)	0.86(0.35–2.14)
	Specialist	10(11)	Ref	Ref	Ref	Ref
4 or more days	Standard	70(60)	0.79(0.40–1.55)	0.61(0.29–1.31)	0.61(0.27–1.34)	0.61(0.28–1.35)
	Group	33(28)	0.73(0.35–1.51)	0.63(0.27–1.42)	0.63(0.27–1.43)	0.72(0.31–1.65)
	Specialist	14(12)	Ref	Ref	Ref	Ref

* Model 1: Adjusted for demographics ethnicity, age, parity, IMD score, any social and medical risk factors at booking and onset of labour.

** Model 2: Model 1 + Adjustment for place of antenatal care (community or hospital).

*** Model 3: Model 2 + Adjustment for service provider attended (A or B).

#### Analysis 2- place of antenatal care

Table 15 shows no significant relationship between service use outcomes and place of antenatal care.

**Table 15:** Women’s service use in relation to place of antenatal care.

Service use	Place of antenatal care	Number of women (%)	Unadjusted RR	Model 1	Model 2	Model 3
			(95% CI)	Adjusted RR (95% CI) *	Adjusted RR (95% CI) **	Adjusted RR (95% CI) ***
1 or more antenatal admissions	Hospital	102(68)	**2.00(1.37–2.91)**	1.38(0.91–2.09)	1.46(0.94–2.26)	1.00(0.61–1.64)
	Community	49(32)	Ref	Ref	Ref	Ref
Length of postnatal stay:						
0–1 day	Hospital	199(52)	Ref	Ref	Ref	Ref
	Community	180(48)	Ref	Ref	Ref	Ref
2 days	Hospital	105(58)	1.25(0.87–1.80	1.15(0.77–1.71)	1.09(0.72–1.67)	0.93(0.58–1.48)
	Community	75(42)	Ref	Ref	Ref	Ref
3 days	Hospital	49(52)	0.97(0.62–1.53)	0.83(0.50–1.37)	0.83(0.49–1.40)	0.83(0.47–1.46)
	Community	45(48)	Ref	Ref	Ref	Ref
4 or more days	Hospital	65(56)	1.08(0.71–1.65)	0.85(0.60–1.52)	0.98(0.60–1.61)	1.14(0.68–1.93)
	Community	52(44)	Ref	Ref	Ref	Ref

* Model 1: Adjusted for demographics ethnicity, age, parity, IMD score, social risk and medical risk factors.

** Model 2: Model 1 plus adjusted for model of care.

*** Model 3: Model 2 plus Adjusted for service provider attended.

### Summary of findings

These findings are summarised in Table 16 below, showing the significant findings in relation to either the model of care received, the place of antenatal care, and the service provider. Characteristics of women at disproportionate risk are also presented.

**Table 16:** Overview of outcomes.

**Outcome variable**	**Characteristics of women at disproportionate risk when adjusting (**S2 Appendix)	**Significant effect of specialist model of care**	**Significant effect of hospital based antenatal care**	**Significant effect of service provider**
**Maternal birth outcomes**				
Elective caesarean section	High medical risk	=	=	B ↑
Emergency caesarean section	Primiparous	=	=	=
	High medical risk			
Instrumental delivery	Primiparous	=	=	=
	High medical risk			
Postpartum haemorrhage	Primiparous	=	=	B ↑
	High medical risk			
Massive obstetric haemorrhage	High medical risk	=	=	=
	Social risk factor(s)			
Perineal trauma	Primiparous	=	=	=
Obstetric emergency	Primiparous	=	=	=
	High medical risk			
Epidural/CSE/GA in labour	Primiparous	=	=	B ↑
	High medical risk			
	Over 34 years old			
Opioid in labour	Primiparous	=	=	=
No analgesia or Entonox only in labour	Multiparous	=	=	=
Water for pain relief in labour	High medical risk	↑	=	=
	Increased age			
Monitoring (CTG in labour)	Primiparous	=	=	B ↑
	High medical risk			
Induction of labour	Primiparous	=	↓	=
	High medical risk			
Place of birth- obstetric led	High medical risk	=	=	B ↑
**Neonatal Outcomes**	**Characteristics of women at disproportionate risk when adjusting (**S2 Appendix)	**Significant effect of specialist model of care**	**Significant effect of hospital based antenatal care**	**Significant effect of service**
Premature birth (<37/40weeks)	Primiparous	=	↑	=
Low birthweight (<2500g)	Primiparous	=	↑	=
	High medical risk			
Apgar scores	Social risk factor(s)	=	=	B↓
	Black Caribbean			
Neonatal unit admission	Black African	=	=	=
Stillbirth/neonatal death	Social risk factor(s)	N/A	=	=
Artificially fed infant at discharge	High medical risk	=	=	B ↑
	Black ‘other’ ethnicity			
Skin-to-skin contact	Black Caribbean	↑	=	B ↓
	Social risk factor(s)			
	High medical risk			
**Hospital stay**				
Antenatal admissions	Black Caribbean	=	=	B ↑
	Black ‘other’			
	High medical risk			
Length of postnatal stay	Primiparous	=	=	=
	High medical risk			

↑ = Statistically significant increase (Pr < 0.05).

↓ = Statistically significant decrease (Pr < 0.05).

= No significant relationship detected.

‘A’ and ‘B’ refer to services.

### Subgroup analysis

Outcomes that were associated with a significant relationship to either the model of care received, or the place of antenatal care attended were analysed for the ‘most at risk’ women only. This subgroup included:

- Women with IMD scores within the most deprived 3 deciles and/or
- Not white ethnicity and/or
- Any social risk factor

This subgroup accounted for 593 women, 59.30% of the sample.

#### Analysis 1- model of care

Table 17 shows that of the 593 women with increased social risk, only 7 used water for pain relief in labour. The relationship to model of care was not seen for women with increased social risk. However, for skin-to-skin contact there remained a significant relationship, with women at increased risk who received the specialist model of care being more likely to experience this important bonding practice.

**Table 17:** Subgroup analysis by model of care received.

Outcome	Model of Care	Number of women (%)	Unadjusted RR	Model 1	Model 2	Model 3
			(95% CI)	Adjusted RR (95% CI) *	Adjusted RR (95% CI) **	Adjusted RR (95% CI) ***
Water for pain relief in labour	Standard	3(43)	0.61(0.63–6.05)	0.55(0.41–7.50)	0.64(0.04–8.85)	0.45(0.02–6.98)
	Group	3(43)	1.35(0.12–13.3)	1.61(0.12–20.4)	1.67(0.13–21.3)	1.90(0.14–25.3)
	Specialist	1(14)	Ref	Ref	Ref	Ref
Skin-to-skin contact	Standard	216(59)	**0.43(0.21–0.89)**	**0.28(0.13–0.64)**	**0.37(0.16–0.85)**	**0.32(0.13–0.77)**
	Group	97(26)	**0.39(0.18–0.85)**	**0.28(0.12–0.65)**	**0.29(0.12–0.69)**	**0.36(0.14–0.89)**
	Specialist	54(15)	Ref	Ref	Ref	Ref

* Model 1: Adjusted for demographics ethnicity, age, parity, IMD score, any social and medical risk factors at booking and onset of labour.

** Model 2: Model 1 + Adjustment for place of antenatal care (community or hospital).

*** Model 3: Model 2 + Adjustment for service provider attended (A or B).

#### Analysis 2- place of antenatal care

The significant outcomes associated with place of antenatal care were also analysed for the ‘most at risk’ subgroup, see Table 18. When the rate of induction of labour was analysed for the subgroup the relationship between hospital and increased induction was no longer significant suggesting that the women with less social risk attending hospital antenatal care are more likely to experience induction of labour. For the whole sample, women attending the hospital for their antenatal care are more likely to experience preterm birth compared to those attending community based antenatal care (RR2.38 CI 1.32–4.27), but the risk increases for the ‘most at risk’ subgroup (RR 3.11 CI1.49–6.50). The relationship between hospital-based antenatal care and low birthweight remained significant but did not increase for the subgroup.

**Table 18:** Subgroup analysis by place of antenatal care attended.

Outcome	Place of antenatal care	Number of women (%)	Unadjusted RR	Model 1	Model 2	Model 3
			(95% CI)	Adjusted RR (95% CI) *	Adjusted RR (95% CI) **	Adjusted RR (95% CI) ***
Induction of labour	Hospital	118(52)	0.86(0.60–1.23)	0.92(0.63–1.34)	0.88(0.60–1.31)	0.87(0.55–1.37)
	Community	104(46)	Ref	Ref	Ref	Ref
Preterm Birth	Hospital	45(76)	**2.85(1.52–5.35)**	**3.15(1.62–6.15)**	**3.24(1.63–6.42)**	**3.11(1.49–6.50)**
	Community	14(24)	Ref	Ref	Ref	Ref
Low birthweight	Hospital	35(67)	1.76(0.95–3.23)	**2.21(1.14–4.30)**	**2.10(1.06–4.15)**	**2.09(1.00–4.34)**
	Community	17(33)	Ref	Ref	Ref	Ref

* Model 1: Adjusted for demographics ethnicity, age, parity, IMD score, social risk and medical risk factors.

** Model 2: Model 1 plus adjusted for model of care.

*** Model 3: Model 2 plus Adjusted for service provider attended.

Further findings of the wider evaluation can be found at https://www.project20.uk.

## Discussion

Although there is encouraging evidence that continuity of midwifery care improves birth outcomes [40], the mechanisms underlying these improvements, and the impact on clinical outcomes for women with social risk factors is largely absent in the literature, prompting a need for this research. The two specialist models of care evaluated in this study were similar in that they both provided continuity of midwifery care to women with low socioeconomic status and social risk factors. The main differences between the models is that one was based within a local community health service ‘hub’ and the other within a large teaching hospital. These differences allowed for the exploration of mechanisms based not only on continuity of care but also the impact of place-based care.

To summarise, no significant differences were found between the model of care and majority of the maternal outcomes. This is important considering women in the specialist model were more likely to have high deprivation scores and more social risk factors, and therefore more likely to experience poor maternal birth outcomes such as caesarean section and obstetric emergencies [4, 27, 68, 69]. Therefore the specialist model of care, and in some cases the group practice model, appear to offer protection against the poorer outcomes that might be expected for these women under standard care’. The specialist model of care was associated with improved birth outcomes such as skin to skin contact after birth and the use of water as pain relief in labour. Interestingly, different relationships were found between the place of antenatal care and neonatal outcomes such as premature birth and low birth weight. Table 16 presents specific demographics that put women at disproportionate risk of each outcome. These were often related to race, age, parity, deprivation score, medical risk status, and social risk factors, and were used to develop a subgroup to further analyse the significant findings. Social risk factors were associated with an increase in stillbirth and neonatal death in the adjusted models. Given the small numbers and findings for preterm births and neonatal unit admissions this warrants further investigation of the relationship between place of antenatal care and stillbirth or neonatal death in future research. The subgroup analysis found that for most outcomes there was little difference in effect compared to the whole cohort, but when preterm birth was analysed for the sub-group, women attending the hospital-based model who were at increased social risk were even more likely to have premature birth than those in the full analysis.

The variation seen between different aspects of maternity care, women’s demographics and their outcomes highlights the individual nature of pregnancy and birth. There is no ‘one size fits all’ approach to improving all outcomes for all women; care must be tailored to meet these individual needs and a starting point for this is continuity of care through which women’s needs can be realised. The differences seen between the findings of this study and the Cochrane review of midwife led models of care [40] can be largely explained by the population being analysed and the place of care. Where some of the trials included in the review excluded women with medical risk factors and substance abuse, others were based in the hospital setting. A subgroup analysis of women with social risk factors and place of antenatal care would be a useful contribution to the review.

As established in the introduction, women with low socioeconomic status and social risk factors are more susceptible to poor infant birth outcomes, including preterm birth (birth before 37 weeks’ gestation). Despite efforts to decrease its prevalence, improve clinical management and reduce infant morbidity and mortality, preterm birth rates continue to rise in most countries [70]. This is an important outcome and indicator for health over the life course with many preterm neonates going on to have significant developmental delay, learning disabilities, visual and hearing problems, chronic lung disease as well as other health implications [71, 72]. These factors lead to increased costs to health services, the economy and the broader society [70]. There are many predisposing, and often intersecting, factors associated with preterm birth that are important to bear in mind as we attempt to propose specific mechanisms that reduce preterm birth rates for women who are accessing care in the community setting. These factors include; infection, social stress, intimate partner violence, non-Caucasian ethnicity, young or advanced age, previous preterm birth, short inter-pregnancy intervals, nutritional deficiencies, cervical procedures, underlying medical conditions, smoking and alcohol consumption, and pollution exposure [31, 73–75]. As discussed in the introduction, women from Black and minority ethnic groups and those with social risk factors are more likely to be living in poverty, experiencing multiple health issues and have poorer experiences of healthcare services driven by discrimination than their white counterparts [76, 77]. Women from Black and minority ethnic groups, who are at increased risk of preterm birth, have described the effect of ‘weathering’ in relation to accessing medical care. First coined by Geronimus [78], ‘weathering’ posits that Black women’s health deteriorates in early adulthood as a result of the cumulative effects of socioeconomic disadvantage. The theory has been widely tested and supported through analysis of health inequalities seen in pregnancy outcomes, excess mortality, disability and mental health [79–83]. Geronimus’ theory led the way to phenomena such as the allostatic load [84], epigenetics [85], and telomere shortening (a marker of cellular aging), all of which have been associated with preterm birth and the cumulative effect of stress and exposure to discrimination on the body [86–88]. The question is then, how can maternity care acknowledge and aim to reduce the effect of these stressors, not only to improve pregnancy outcomes but also to break the cycle of socioeconomic disadvantage and its associated health inequalities?

In addition to the Cochrane reviews of models of care and interventions to reduce preterm birth [40, 89], a systematic review and meta-analysis of models of antenatal care designed to reduce preterm birth [61] found that women randomised to midwife-led continuity models of antenatal care were less likely to experience preterm birth regardless of their medical risk factors. The evidence base concludes that although alternative models of antenatal care can be effective in reducing preterm birth, the mechanisms, including the effect of place of antenatal care remain unknown.

**Fig 2:**
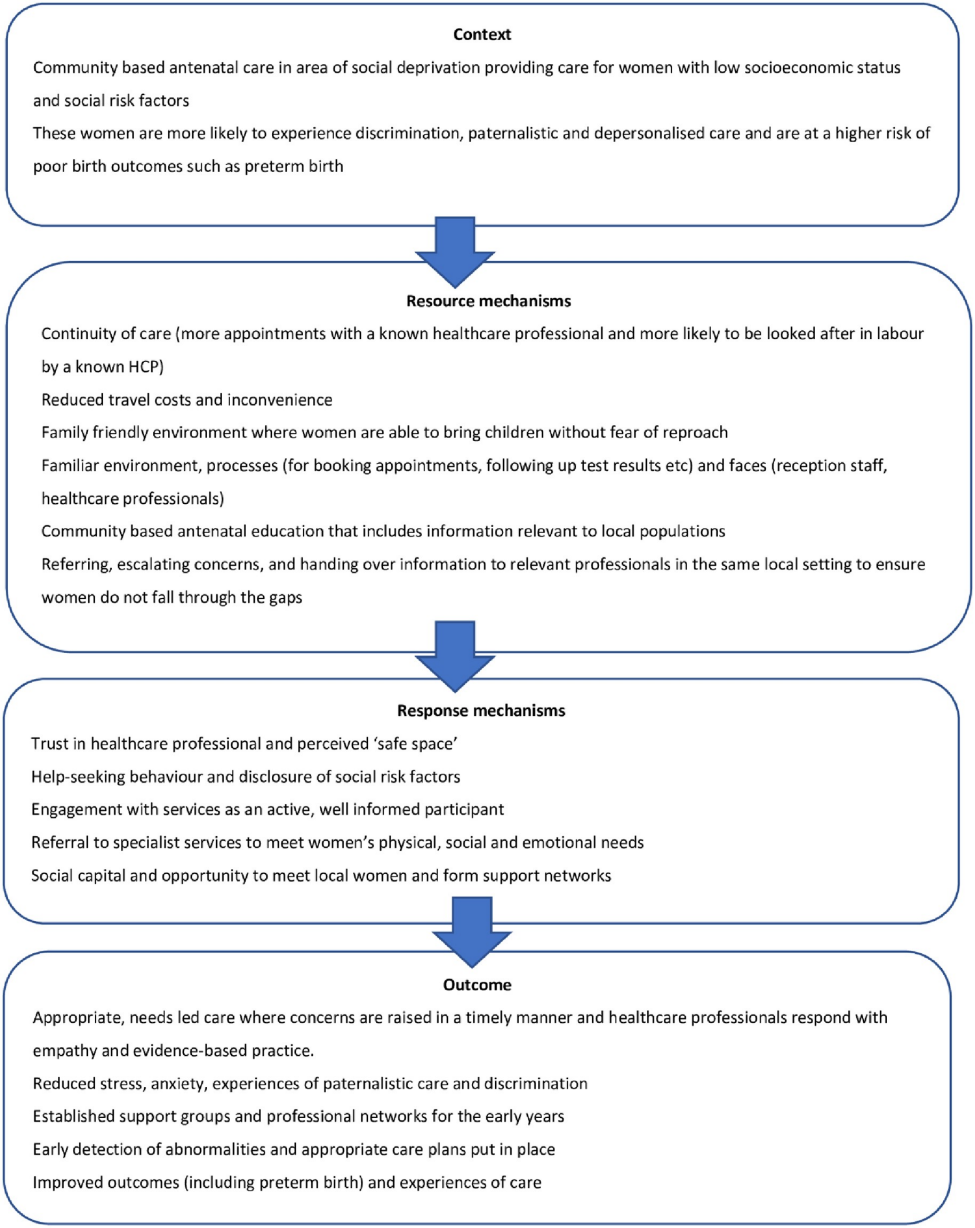
CMO configuration to reduce inequalities seen in preterm birth rates.

The hospital environment has long been associated with increased stress, waiting times, unfamiliarity, fragmentation and impersonal care [30, 90–92]. When the stressful effects of the hospital environment are compounded by paternalistic care, a lack of choice and perceived stigma and discrimination often described by Black and minority ethnic women and those with social risk factors [28, 30, 93], poor outcomes and experiences can be exacerbated. Acknowledgement of the effect of environment is seen in recent policy with the NHS long term plan [94] and five year forward view [95] emphasising the value of expanding community-based health services on people’s health, help-seeking behaviours and pressures on the wider service. The concept of women handing over control and choice to the healthcare professional was also described in Ebert et al.’s [96] qualitative work with socially disadvantaged women in Australia. The study concluded with the recommendation to step away from medically focused maternity care environments in order to create ‘safe spaces’ for women. Although the place of care is not discussed in Ebert et al.’s study, care set within women’s local communities could be a solution to protecting women from the medicalised hospital environment where they feel disempowered and silenced. Supporting this theory, focus group’s with midwives providing the specialist models of care evaluated in this study described how midwives working in the community setting were more sensitive to women’s wider needs, able to act quickly on abnormal findings or concerns and had increased knowledge of local support available. Another protective factor of community-based care to consider is that of ‘ethnic maintenance’, describing the social connections and cultural norms that multi-cultural communities provide [97]. These combined insights contribute to the theory that care based within the community setting may be perceived by women to align more closely with their needs as the service has ‘come to them’. These insights are presented in ‘context + mechanism = outcome’ format in Fig 2:

This study also found significant relationships between the specialist model of care and the increased number of women using water for pain relief and practising skin-to-skin contact with their baby shortly after birth. The use of water for non-pharmacological pain relief in labour is associated with a reduction in the duration of labour and use of epidural anaesthesia, fewer interventions and transfer to obstetric units in labour, and no adverse outcomes [98, 99]. Skin-to-skin contact, sometimes referred to as ‘kangaroo care’, can be defined as ‘placing a naked infant onto the bare chest of the mother’ [100] the benefits of which include improved adaptation to extrauterine life, stimulation of the digestive system and hormone release leading to improved feeding, protection against infection, reduced cortisol levels, and parent-infant bonding [100–103]. A recently published trial of a specialist continuity model of care for women at risk of preterm birth found those women randomised to the intervention were significantly more likely to have skin-to-skin contact after birth and to have it for a longer time [104]. Although the underlying mechanisms for these outcomes remain unclear and warrant further research, the phenomenon could be explained using Lipsky’s [105] street level bureaucracy theory in that when midwives know women and are invested in them and their outcomes, they are more likely to provide gold standard practice. This was referred to in the focus groups with the midwives providing the specialist models of care [106] and is summed up well in the following quote: *‘‘I think we also have that like emotional insight as well… I feel like we*, *as a team*, *we are quite invested in our women*, *and we do do a lot for them and I think*, *when you have that investment in someone that you want to push for them*, *and you want their outcome to be good’*.

### Strenghts and limitations

As with all cohort studies, particularly those using records that were not designed for the purpose of the study, there are limitations such as potential poor data quality and differential loss to follow up [107]. In an attempt to minimise these limitations we took a prospective approach to data collection and analysed the demographic data of women who did not go on to give birth at the study sites. The analysis of maternal and neonatal birth outcomes took a multinominal model approach to separately analyse the effect of numerous potentially confounding factors such as demographics, the place of care, model of care received, and service attended. Limited by the use of the IMD score as the only measure of deprivation available to the researchers, future research would be strengthened by analysing other potentially confounding factors and measures of deprivation and social exclusion such as income, occupation, and women’s support networks, as well as measures of perceived discrimination that has been linked to poorer maternal outcomes in the US [108]. An analysis of maternal deaths in the UK [16] found medical comorbidities to be a main driver of maternal death. The analysis did not include mental health as a comorbidity but more recent research [109] highlighted it’s significant impact on maternal morbidity. Although this study adjusted for high medical risk factors, data on the number and nature of medical conditions was not collected and should be addressed in future research to identify the underlying mechanisms and how health services can better meets the needs of those with physical and mental health comorbidities. Differences in the models of care and place of antenatal care such as working practises, environment and midwives characteristics could also refine our understanding of the causal mechanisms leading to improved outcomes. The relatively small numbers in each quantitative data group should be taken into consideration due to the significant amount of multiple testing required to establish the separate effects of the potentially confounding factors. This presents a potential limitation as the use of multiple testing can result in a substantial change statistical power, reducing the probability of detecting effects when they do exist and increasing the probability of finding significant differences by chance [110]. This could be overcome in future research using larger sample sizes to test the apparent mitigating effects of the specialist models and community antenatal care on health inequalities.

The generalisability of the findings is limited by the urban location of both specialist models of care evaluated. This is particularly significant when reflecting on the outcomes relating to place-based care- what may have significant outcomes in a densely populated, inner-city, multicultural community, may yield very different results elsewhere. Research is needed to test the generalisability of the findings to rural and other community settings. The wider evaluation of the models of care described in this study will give insight into the underlying mechanisms for the outcomes reported, as well as further detail into women’s access and engagement and the quality of the relational continuity they experienced.

## Conclusion

The findings presented in this study highlight how different aspects of maternity care can lead to different outcomes dependant on women’s specific demographics and circumstances. It reveals insight into the complexity of the mechanisms underpinning specialist models of care and how they could lead to a narrowing of inequalities in pregnancy related outcomes. Unpicking these mechanisms allows the formation of new hypotheses to test around place-based care, its impact on neonatal outcomes, and the development of maternity services that aim to reduce health inequalities for local populations. The mechanisms listed in the CMO configuration could be used as a framework for those developing maternity services, with the recommendation to audit outcomes to enable a greater understanding of how they might work in different contexts. Although the findings support the policy drive to scale up models of maternity care that offer continuity to those at increased risk, this study reveals that continuity alone is not a panacea for all pregnancy and birth inequalities. Other aspects of maternity care such as where it is placed, the level of choice and control it provides women with, and how autonomously midwives can practice need to be carefully considered by those implementing services.

## Supporting information

S1 Appendix Definitions. (DOCX)

S2 Appendix Data analysis. (DOCX)
