# Supplementary material for: Project20: Does continuity of care and community-based antenatal care improve maternal and neonatal birth outcomes for women with social risk factors? A prospective, observational study
Source: PLoS One. 2021 May 4;16(5):e0250947. doi: 10.1371/journal.pone.0250947 (PMC8096106; doi:10.1371/journal.pone.0250947)
Supplement: S1 Appendix — (DOCX) [file pone.0250947.s001.docx]

# S1 Appendix: Definitions

Table 1: Definitions of the different models of care received by women at the two service providers evaluated

| **Service A** | **Standard Care:** Depending on medical risk factors women receive antenatal and postnatal care as set by NICE guidance ^1^ in either the community setting or hospital setting. For low-risk women care is usually provided by a community midwife in a GP surgery or local children’s centre. For women at high medical risk care is provided at the hospital and shared between midwives and obstetricians. Although women may be assigned a ‘named’ healthcare professional, there is no emphasis on the provision of continuity of care.  **Group Practice:** Women are seen in either the community or hospital setting depending on their medical risk status. There is an aim to provide antenatal and postnatal continuity of care. Women have a named midwife who aims to see them for the majority of their antenatal and postnatal appointments. Intrapartum care is covered by the hospitals labour ward or birth centre staff. Women planning a homebirth will be looked after by on-call midwives, but this may be from a team not known to the woman.  **Specialist model: - Community-based Model [CBM]**  A team of 6 midwives provide continuity of care to women located in an area of social deprivation. Not all women under their care will have social risk factors. Each woman is assigned a named midwife who coordinates all care, multi-disciplinary communication, and referrals. The named midwife aims to provide the vast majority of clinical care, with others in the team providing care when s/he is not on duty. The midwives are based in a local community health centre and offer antenatal, intrapartum, and postnatal care in the home, community, or hospital setting. |
| --- | --- |
| **Service B** | **Standard Care:** Depending on medical risk factors women receive antenatal and postnatal care as set by NICE guidance ^1^. If women do not live within the geographical catchment areas of the group practices (see definition below), care is usually provided at the hospital and shared between midwives and obstetricians. Although women may be assigned a ‘named’ healthcare professional, there is no emphasis on the provision of continuity of care.  **Group practice** Women are seen in different settings depending on their medical risk status but there is an aim to provide antenatal and postnatal continuity of care. For the majority of women who live within the hospital’s geographical catchment area, care is provided in the community setting, often out of children’s centres to prevent women from having to travel to the hospital for appointments. Postnatal care is provided at home and in postnatal clinics in the community. Women have a named midwife who aims to see them for the majority of their antenatal and postnatal appointments. Intrapartum care is covered by the hospitals labour ward or birth centre staff. Women planning a homebirth are looked after by a team of midwives providing on call care, with the aim for the midwives to have met the woman before.  **Specialist model: - Hospital-based Model [HBM]**  A team of 6 midwives provide continuity of care to women with social risk factors only. Women with one or more significant social risk factors (see Appendix A for inclusion criteria) are referred to the team and assigned a named midwife who coordinates all care, multi-disciplinary communication, and referrals. The named midwife aims to provide the vast majority of clinical care, with others in the team providing care when she/he is not on duty. The midwives are based at the hospital site and offer antenatal, intrapartum, and postnatal care in the home or hospital setting. |

Table 2: Outcome variable definitions

| **Term** | **Definition** |
| --- | --- |
|  |  |
| Deprivation score | A composite measure using routine data from the seven domains of deprivation to identify the most disadvantaged areas in England, UK ^2^ |
|  |  |
| Ethnicity | Using the ONS 18+ categories of ethnicity |
|  |  |
| Medical risk status | As recorded by healthcare professional entering routinely collected maternity record data |
| Place of birth | Labourward: an NHS clinical location in which care is provided by a team, with obstetricians taking primary professional responsibility for women at high risk of complications during labour and birth. Midwives offer care to all women in an OU, whether or not they are considered at high or low risk, and take primary responsibility for women with straightforward pregnancies during labour and birth.  Birth Centre: an NHS clinical location offering care to women with straightforward pregnancies during labour and birth in which midwives take primary professional responsibility for care.  Home: Birth planned in a non-NHS setting, usually the woman’s residence, facilitated by midwives. |
|  |  |
| Length of postnatal stay | Inpatient stay immediately after birth |
|  |  |
| Induction of labour | The process of artificially stimulating the uterus to start labour by administering prostaglandins, oxytocin, or artificially rupturing membranes. Does not include cervical sweeps. |
| Estimated blood loss | Estimated blood loss at birth or in the immediate postnatal period as recorded by healthcare professional entering routinely collected maternity record data. |
| Obstetric emergency | For the purpose of this research obstetric emergency refers to recorded antenatal or postpartum haemorrhage, shoulder dystocia, cord prolapse, neonatal resuscitation, eclamptic seizure and undiagnosed breech and/or twin birth. |
| Maternal death | The death of a woman while pregnant or within 42 days of termination of pregnancy, irrespective of the cause of death. |
| Stillbirth  Neonatal death | A baby delivered with no signs of life known to have died after 24 completed weeks of pregnancy. Intrauterine fetal death refers to babies with no signs of life in utero.  A baby born at any time during the pregnancy who lives, even briefly, but dies within four weeks of being born. |
| Apgar scores | A measure of the physical condition of a newborn infant. It is obtained by adding points (2, 1, or 0) for heart rate, respiratory effort, muscle tone, response to stimulation, and skin coloration; a score of ten represents the best possible condition. |
|  |  |
| Skin-to-skin | Skin-to-skin contact is usually referred to as the practice where a baby is dried and laid directly on their mother's bare chest after birth, both of them covered in a warm blanket and left for at least an hour or until after the first feed. |
|  |  |
|  |  |
