# Supplementary material for: Project20: Does continuity of care and community-based antenatal care improve maternal and neonatal birth outcomes for women with social risk factors? A prospective, observational study
Source: PLoS One. 2021 May 4;16(5):e0250947. doi: 10.1371/journal.pone.0250947 (PMC8096106; doi:10.1371/journal.pone.0250947)
Supplement: S2 Appendix — (DOCX) [file pone.0250947.s002.docx]

S2 Appendix: Supplementary data

| Table 1: Missing data reason across each service | | | |  |
| --- | --- | --- | --- | --- |
| **Reason outcome  data missing** | **Service A *n(%)***  ***Total missing data = 95*** | **Service B *n(%)***  ***Total missing data= 106*** | ***TOTAL n(%)***  ***Total missing data=201*** | **X^2^ p value** |
| Moved out of area  Care at other hospital  Miscarriage/TOP  Unknown | *8(8)*  *18(20)*  *35(37)*  *34(36)* | *21(20)*  *23(22)*  *37(35)*  *25(24)* | *29(14)*  *41(20)*  *72(36)*  *59(29)* | *Pr 0.063* |

| Table 2: Missing pregnancy and birth outcome data by deprivation | | | | | |  |
| --- | --- | --- | --- | --- | --- | --- |
| **Reason outcome  data missing** | **Least deprived (7^th^, 8^th^, 9^th^ +10^th^)**  **Total=49** | **5^th^ and 6^th^ deciles**  **Total=42** | **3^rd^ and 4^th^ deciles**  **Total=77** | **Most deprived (1^st^ +2^nd^ deciles)**  **Total=33** | **Total n(%)**  **N=201** | **X^2^ p value** |
| Moved out of area  Care at other hospital  Miscarriage/TOP  Unknown | 12(24)  12(24)  11(22)  14(29) | 5(12)  9(21)  20(48)  8(19) | 10(13)  15(19)  28(36)  24(31) | 2(6)  5(15)  13(39)  13(39) | 29(14)  41(20)  72(36)  59(29) | Pr 0.154 |

Table 3: Maternity care received, and social risk factors recorded by deprivation score

| **Maternity care** | **Least deprived (7^th^, 8^th^, 9^th^ +10^th^)** | **5^th^ and 6^th^ deciles** | **3^rd^ and 4^th^ deciles** | **Most deprived (1^st^ +2^nd^ deciles)** | **Total n(%)** | **X^2^ p value** |
| --- | --- | --- | --- | --- | --- | --- |
| **Model of care**  Standard Care  Group Practice  Specialist  Private Care  **Place of antenatal care***  Hospital based  Community based | **Total=149**  81(54)  43(29)  3(2)  22(15)  **Total=127**  82(65)  45(35) | **Total=158**  98(62)  41(26)  16(10)  3(2)  **Total=155**  91(59)  64(41) | **Total=286**  163(57)  85(30)  34(12)  4(1)  **Total=282**  134(48)  148(52) | **Total=206**  127(62)  52(25)  27(13)  0(0)  **Total=206**  111(54)  95(46) | **N=799**  469(58)  221(28)  80(10)  29(4)  **Total=770**  418(54)  352(46) | **Pr 0.000**  **Pr0.008** |
| **Risk Factors** | Least deprived (7^th^, 8^th^, 9^th^ +10^th^) | 5^th^ and 6^th^ deciles | 3^rd^ and 4^th^ deciles | Most deprived (1^st^ +2^nd^ deciles) | Total n(%) | X^2^ p value |
| Social Risk Factors  None  One  Two  Three  Four or more | **Total=149**  133(89)  10(7)  5(3)  1(1)  0 | Total=158  132(84)  17(11)  4(3)  2(1)  3(2) | Total=286  212(74)  50(17)  10(4)  10(4)  4(1) | Total=206  139(67)  27(13)  20(10)  8(4)  12(6) | **N=799**  616(77)  104(13)  39(5)  21(3)  19(2) | Pr 0.000 |
| Medical Risk  High at booking  High at onset of labour | 37(25)  62 (42) | 46(29)  79(50) | 85(30)  138(48) | 56(27)  96(47) | 224(28)  375(47) | Pr 0.743  Pr 0.475 |

*Excludes private care

## Adjusted and unadjusted data analysis tables

Table 1: Model of care received by level of deprivation

Table 2: Place of antenatal care by level of deprivation

Table 3: Mode of birth in relation to the model of care received

Table 4: Postpartum haemorrhage in relation to the model of care received

Table 5: Massive obstetric haemorrhage in relation to the model of care received

Table 6: Perineal trauma requiring stuturing in relation to the model of care received

Table 7: Obstetric emergency in relation to the model of care received

Table 8: Mode of birth in relation to place of antenatal care

Table 9: Postpartum haemorrhage in relation to place of antenatal care

Table 10: Massive obstetric haemorrhage in relation to place of antenatal care

Table 11: Perineal trauma requiring suturing in relation to place of antenatal care

Table 12: Obstetric emergency in relation to place of antenatal care

Table 13: Analgesia used in labour in relation to model of care

Table 14: CTG monitoring in relation to model of care

Table 15: Induction of labour in relation to model of care

Table 16: Use of analgesia in labour in relation to the place of antenatal care

Table 17: CTG monitoring in labour in relation to the place of antenatal care

Table 18: Induction of labour in relation to the place of antenatal care

Table 19: Place of birth in relation to model of care received

Table

Table 20: Place of birth in relation to place of antenatal care

Table 21:Neonatal outcomes in relation to the model of care received

Table 22: Neonatal outcomes in relation to the place of antenatal care

Table 23: Feeding method and skin-to-skin in relation to the model of care

Table 24:Feeding method and skin-to-skin in relation to place of antenatal care

Table 25:Womens service use in relation to the model of care received

Table 26:Womens service use in relation to the place of antenatal care

Table 27: Subgroup analysis by model of care received

Table 28: Subgroup analysis by place of antenatal care
